# Heart Disease in Women: Unappreciated Challenges, GPER as a New Target

**DOI:** 10.3390/ijms17050760

**Published:** 2016-05-18

**Authors:** Ross D. Feldman

**Affiliations:** Discipline of Medicine, Memorial University of Newfoundland, St. John’s, NL A1B 3V6, Canada; ross.feldman@med.mun.ca; Tel.: +1-709-864-2877; Fax: +1-709-777-8031

**Keywords:** estrogen, aldosterone, G protein-coupled estrogen receptor (GPER), hypertension, dyslipidemia, atherosclerosis

## Abstract

Heart disease in women remains underappreciated, underdiagnosed and undertreated. Further, although we are starting to understand some of the social and behavioral determinants for this, the biological basis for the increased rate of rise in atherosclerosis risk in women after menopause remains very poorly understand. In this review we will outline the scope of the clinical issues related to heart disease in women, the emerging findings regarding the biological basis underlying the increased prevalence of atherosclerotic risk factors in postmenopausal women (*vs.* men) and the role of the G protein-coupled estrogen receptor (GPER) and its genetic regulation as a determinant of these sex-specific risks. GPER is a recently appreciated GPCR that mediates the rapid effects of estrogen and aldosterone. Recent studies have identified that GPER activation regulates both blood pressure. We have shown that regulation of GPER function via expression of a hypofunctional GPER genetic variant is an important determinant of blood pressure and risk of hypertension in women. Further, our most recent studies have identified that GPER activation is an important regulator of low density lipoprotein (LDL) receptor metabolism and that expression of the hypofunctional GPER genetic variant is an important contributor to the development of hypercholesterolemia in women. GPER appears to be an important determinant of the two major risk factors for coronary artery disease-blood pressure and LDL cholesterol. Further, the importance of this mechanism appears to be greater in women. Thus, the appreciation of the role of GPER function as a determinant of the progression of atherosclerotic disease may be important both in our understanding of cardiometabolic function but also in opening the way to greater appreciation of the sex-specific regulation of atherosclerotic risk factors.

## 1. Introduction

The risk of heart disease in women remains underappreciated despite concerted efforts by a range of health care agencies and non-governmental organizations. There remains a wide-spread belief by both health care practitioners and cardiovascular scientists that women are less likely to have either heart disease or heart disease risk factors than men, so any related concerns are misplaced. Nothing could be farther from the truth. We now know that (i) women (after menopause) have a faster rise in cardiovascular (CV) risk than men (ii) that women with risk factors for heart disease are less likely to be treated appropriately (iii) that women with heart disease are less likely to be diagnosed, less likely to be treated and more likely to die of their heart disease or of complications of revascularization (reviewed in [1]). Yet despite these inconvenient truths, the biological basis for sex-specific risks of heart disease in women remains unclear.

## 2. Heart Disease Morbidity/Mortality in Women

Heart disease is an equal opportunity phenomenon. Women are as likely as men to die of heart disease [2]. Additionally, women with heart disease have worse outcomes than men. In hospital mortality following myocardial infarction is more than 50% higher in women than men [3]—even after adjustment for age. Further, in-hospital mortality rates after revascularization are higher in women [4].

The determinants of these risks are multi-factorial. They do include important cultural, social and behavioral factors. As an example, there is important sex and gender differences in the “typical” presentations of symptoms of heart disease differ between genders and that the responses of health care providers to those presentations differ between genders [1]. Additionally, important biological differences in heart disease determinants between sexes are beginning to be appreciated. These include differences in clotting characteristics and differences in the presentation of atherosclerosis (towards smaller vessel disease presentation in women) [5]. Notably, the basis of these genetic differences (beyond the absence or presence of a Y chromosome) has remained largely unstudied.

## 3. Cardiovascular Risk Factors in Women

Although coronary heart disease risk is very low and does not substantially differ between sexes up to age 40, there is a significantly lower risk of heart disease in women *vs.* men through their middle age. Notably, that trend reverses around menopause. Subsequently, the rate of rise in risk is more than 50% higher in women than men, with women catching up to men in their risk of heart disease by age 70 [6]. Further, women have generally worse atherosclerotic risk factor profiles than males. Specifically, they tend to have higher blood pressure (CDC, available online: http://www.cdc.gov/nchs/data/hus/hus11.pdf), higher concentrations of low density lipoprotein (LDL) cholesterol [7] and are more likely to present with multiple atherosclerotic risk factors [8]. Although the increase in BMI seen in women appears to be more dependent on age than on menopause, there is an increase in abdominal subcutaneous and visceral fat with menopause, paralleling the increased prevalence of the metabolic syndrome and ultimately diabetes mellitus [9]. Notably, most studies including the Women’s Health Initiative (WHI) have shown that postmenopausal hormone therapy is associated with reduced abdominal obesity and the development of type II Diabetes Mellitus [10]. As is the case for the increased risk of complications of atherosclerotic disease in women, the biological basis for the increase in the prevalence/severity of cardiovascular risk factors in women post-menopause remains unclear.

## 4. The Increase in Postmenopausal CV Risk in Women Is More than just a Lack of Estrogen

The differences in circulating estrogen levels between men and women were widely thought of as the basis for the lower pre-menopausal heart disease risk in women. Similarly lack of estrogen was widely believed to be the key factor accounting for the rapid acceleration in CV risk post-menopause. These beliefs were supported by decades of study based on animal models and observational studies in humans of the cardiovascular effects (mostly beneficial) of estrogen [11]. However, the strength of those beliefs was shaken by the lack of benefit of post-menopausal estrogen therapy seen in major randomized clinical trials and the suggestion that at least in the short term, post-menopausal estrogen treatment was associated with an increased risk of atherosclerotic/thrombotic events [12]. Further, lost in this discussion was the observation that after menopause the rate of the rise in risk of heart disease is higher in women [6]—suggesting that in this population we should be looking for those factors accounting for the *increase* in risk for heart disease (not just for the basis for the loss in cardioprotection). We would posit that the elucidation of these issues will require a focus not on changes in estrogen hormone levels but a greater appreciation of the cellular basis of estrogen effects—specifically a greater appreciation of the diversity of the consequence of estrogen signaling depending on the receptor system through which it is acting.

## 5. Estrogen Signaling Pathways

Much of what we know about the biological basis for the actions of estrogens is based on the activation of classic estrogen receptors (ER) [11]. These receptors (ERα, ERβ and several splice variants) primarily reside in cytoplasmic and nuclear locations. Upon estrogen binding to ERs they dissociate from their chaperone protein (HSP-90) dimerize and translocate to the nucleus where they regulate pathways of transcriptional regulation [13]. However, it has been appreciated for more than 75 years that some actions of estrogen (especially the cardiovascular effects) occurred over time courses too rapid for those requiring transcription and translation [14]. Some of these effects have been determined to be linked to activation of membrane-associated ERs—especially those linked to the cardiovascular effects of estrogens [15]. However, this was not the case for all. The basis for these non-ER rapid effects of estrogen was elucidated in the early 2000’s-based on the identification of an up-till-then orphan G protein coupled receptor—initially called GPR30 but now better known as GPER [16].

GPER is widely expressed in reproductive tissues but as well ubiquitously—in neural, endocrine, cardiovascular, renal, metabolic (liver, fat) tissues and in immune cells [17]. Notably, GPER activation has a relatively small role mediating the reproductive effects of estrogens. However, its importance as a regulator of neural, neuroendocrine, endocrine and immune functions is being increasingly appreciated [16]. In the cardiovascular systems, GPER’s effects are related to (i) vasorelaxation [18,19] and consequently reducing peripheral resistance and blood pressure [18,20] (ii) inhibiting cell growth in cardiovascular tissues [21,22,23,24] or stimulating programmed cell death [24,25] and (iii) regulation of hepatic cholesterol metabolism [26].

## 6. GPER Is More than Just an Estrogen Receptor

Subsequent to the demonstration that GPER was activated by estradiol, several synthetic ligands including the prototype GPER agonist, G1 and antagonists G1 and G15 were developed which have helped propel research in the field [27]. Additionally, a range of estrogens including estrone and estriol as well as estrogen metabolites including 2-hydroxy estradiol and 2-methoxy estradiol were shown to be receptor ligands, based on binding and/or functional studies (reviewed in [16]). In addition, several selective estrogen receptor modulators including raloxifene and tamoxifen have GPER agonist effects [16]. The previously thought-to-be selective ER antagonist, ICI 182,780 has also been shown to bind to GPER [28]. Further, a growing range of phytoestrogens and xenoestrogens has been shown to have generally GPER agonist effects [16].

More contentious has been the demonstration that estrogen is not the sole physiological hormone that acts via a GPER-dependent pathway. Aldosterone has been shown to have a very high affinity for mediating GPER-dependent effects with potency 10–100 times that of estradiol [29]. GPER-dependent aldosterone effects have been shown in vascular smooth muscle [29] and endothelial cells—where aldosterone’s endothelium-dependent vasodilator effects have been shown to be GPER-dependent [24], in breast cancer cells [30], in cardiomyocytes [31], in cardiac vagal neurons [32], in renal connecting tubules [33]. Notably two recent studies have failed to demonstrate the ability of aldosterone to displace [^3^H] estradiol in radioligand binding to a putative GPER [30,34]. However, whether this means that the effect of aldosterone to activate GPER is indirect (via activation of an associated receptor) or reflects the nonspecificity of current receptor binding methods characterizing GPER binding remains a point of contention.

## 7. Vascular Actions of GPER

Studies of the cardiovascular effects of GPER have mostly focused on its effects on vascular reactivity. However, GPER activation in animal models has been shown to regulate ischemic responses both in the myocardium [35,36] and with cerebrovascular injury [37]. Notably, in the latter study pretreatment of male mice with the GPER agonist, G1 prior to inducing cerebrovascular ischemia worsened recovery from ischemia-reperfusion injury, an effect blocked by the GPER antagonist G15, whereas treatment with G15 had a protective effect. In contrast, treatment of ovariectomized females with G1 improved neurological outcomes following cerebrovascular ischemia, but these effects were not observed in ovary-intact females. The basis for these sex-dependent effects remains to be elucidated.

GPER activation lowers blood pressure both acutely [18] and chronically [20]. Genetic deletion of GPER has been associated with sex-specific increases in blood pressure [38], although the generalizability of this finding has not been universal among the other GPER genetic deletion mouse models [39]. These studies in aggregate have suggested a potential role for GPER as a determinant of human blood pressure control. However the significance of these findings in the chronic regulation of blood pressure in humans remained to be determined.

To examine the importance of GPER-mediated regulation of vascular reactivity in blood pressure control in humans we have utilized a population genetics approach-similar to the strategy we used to demonstrate the significance of isoform-specific regulation of adenylyl cyclase VI [40,41]. This approach is predicated on the ability to identify a missense genetic variant of the gene in question, determine its functionality (whether hypo- or hyper-functional) at a cellular level and to determine whether carrying that genetic variant translates into an alteration of phenotype as predicted by the cellular findings and the expected regulatory effects of the gene product. For GPER, among the 40 single nucleotide polymorphisms identified, three missense single nucleotide variants have been described (GenBank accession No. NM_001039966). Of them, the target for our studies was the P16L GPER variant, which is the most common with an allele frequency of approximately 20% [26,42].

With expression in rat aortic vascular smooth muscle cells—which lose GPER expression in culture [25], the P16L GPER genetic variant demonstrates hyporesponsiveness as compared to the wild type [42]. Assessing GPER-mediated ERK activation and apoptosis, utilizing the GPER agonist, G1, the P16L GPER genetic variant demonstrated reduced effects to stimulate ERK phosphorylation and to stimulate apoptosis compared to wild type GPER—when studied at similar levels of expression [42].

Having demonstrated that this genetic variant of GPER was hypofunctional we further examined whether the expected decrease in GPER vascular functionality in those carrying this polymorphism translated into an altered cardiovascular phenotype. In a predominantly younger, Caucasian cohort, those carrying the hypofunctional P16L GPER genetic variant had higher blood pressures [42]. Notably, when stratified by sex, the increase in blood pressure was only evident in females. Further, to identify whether carrying this genetic variant was a risk for hypertension we determined the allele frequency of the GPER variant in a population of patients with resistant hypertension. We found that women with resistant hypertension were almost twice as likely as men to carry the GPER P16L genetic variant [42]. These data in aggregate would suggest that based on this population genetic approach, GPER regulation is a determinant of blood pressure control in humans- at least in females.

## 8. Metabolic Effects of GPER

Effects of GPER on regulation of metabolic pathways have been demonstrated, specifically related to regulation of insulin secretion [43]. Further, at least one GPER knockout model has been characterized as having higher total cholesterol and low density lipoprotein (LDL) cholesterol [44]. On the other hand, in animal models, estrogen treatment has been associated with increased hepatic LDL receptor expression [45,46]—a key determinant of circulating LDL-cholesterol levels and with decreased proprotein convertase subtilisin/kexin type 9 (PCSK9) activity [47]—the key determinant of LDL receptor degradation and hence of cellular LDL receptor content [48]. However, the receptor mechanism for these phenomena was unclear. To determine the role of GPER we assessed effects of the GPER agonist, G1 in HepG2 liver cells using both the pharmacological antagonism of GPER effects using the antagonist G15 as well as with GPER downregulation using shRNA methods. We showed that GPER stimulated LDL receptor content primarily via inhibition of PCSK9 expression [26].

To determine the functional impact of GPER-mediated regulation of LDL receptor expression in humans we again utilized a population genetics approach—both in the Southwestern Ontario cohort previously utilized in our blood pressure studies but also in a more isolated, genetically homogenous population of Hutterites from small communities in Northern Canada [26]. In both cohorts we showed that females, but not males carrying the P16L GPER genetic variant had higher total and LDL cholesterol concentrations—supporting a role of the extent of GPER responsiveness as a determinant of cholesterol levels.

## 9. Future Directions

The studies to date have identified an important role of GPER activation in the regulation of cardiovascular function especially in women. Important questions remain. In *in vitro* cell systems the balance between GPER *vs.* ER expression has been shown to be an important determinant of the growth stimulatory *vs.* growth inhibitory effects of estrogen [25]. Additionally, there is evidence that with aging and after menopause, GPER expression and function is decreased in females [49]—a least in animal models. The significance of those findings *in vivo*, especially in the setting of vascular injury and particularly in post-menopausal humans remains to be elucidated. However, understanding the *in vivo* implications of altering this balance may be important to our understanding of why women with heart disease are more likely to have complications following revascularization procedures. Moreover, with the appreciation that aldosterone may be an important modulator of estrogen’s actions at GPER, further understanding of the impact of the regulation of aldosterone synthesis on GPER effects remains an important area of further investigation.
